# Regulatory Functions of Natural Killer Cells in Multiple Sclerosis

**DOI:** 10.3389/fimmu.2016.00606

**Published:** 2016-12-19

**Authors:** Catharina C. Gross, Andreas Schulte-Mecklenbeck, Heinz Wiendl, Emanuela Marcenaro, Nicole Kerlero de Rosbo, Antonio Uccelli, Alice Laroni

**Affiliations:** ^1^Department of Neurology, University Hospital Münster, Münster, Germany; ^2^Centre of Excellence for Biomedical Research, University of Genova, Genova, Italy; ^3^Department of Experimental Medicine, University of Genova, Genova, Italy; ^4^Department of Neuroscience, Rehabilitation, Ophthalmology, Genetics, Maternal and Child Health, University of Genova, Genova, Italy; ^5^IRCCS San Martino-IST, Genova, Italy

**Keywords:** natural killer cells, CD56^bright^ NK cells, CD56^dim^ NK cells, regulatory immune cells, innate immune system, multiple sclerosis

## Abstract

There is increasing evidence that natural killer (NK) cells exhibit regulatory features. Among them, CD56^bright^ NK cells have been suggested to play a major role in controlling T cell responses and maintaining homeostasis. Dysfunction in NK cell-mediated regulatory features has been recently described in untreated multiple sclerosis (MS), suggesting a contribution to MS pathogenesis. Moreover, biological disease-modifying treatments effective in MS apparently enhance the frequencies and/or regulatory function of NK cells, further pointing toward an immunoprotective role of NK cells in MS. Here, we summarize the current knowledge on the regulatory functions of NK cells, based on their interactions with other cells belonging to the innate compartment, as well as with adaptive effector cells. We review the more recent data reporting disruption of NK cell/T cell interactions in MS and discuss how disease-modifying treatments for MS affect NK cells.

## Natural Killer (NK) Cells as Controllers of (Auto)Immune Responses

### Regulatory Features of NK Cells

Autoimmune reactivity occurs in every subject, but only 5–10% of humans develop an autoimmune disease (1). Keeping autoreactive cells under control and, thus, preventing them to cause disease is the task of specialized immune cell subsets, called “regulatory” cells. The best characterized regulatory immune cell populations belong to the adaptive immune system (IS) and include regulatory T cells (T_regs_), type-1 regulatory T cells, and regulatory B cells. However, there is increasing evidence that the innate IS also plays an important role in controlling autoreactive cells.

An important population of regulatory immune cells belongs to the natural killer (NK) cells. These, so-called CD56^bright^ NK cells, owe their name to high surface expression of CD56 (also known as neural cell adhesion molecule), are CD16^−/dim^, express the inhibitory receptor NKG2A, and do not express killer cell immunoglobulin-like receptors (KIR). CD56^bright^ NK cells were first considered “immunoregulatory” by Cooper et al., due to increased production of cytokines and reduced cytotoxicity compared to CD56^dim^ NK cells (2).

It is now established that CD56^bright^ NK cells regulate other immune cells belonging to both the innate and adaptive IS. Although most studies on CD56^bright^ NK cell function have been conducted *ex vivo* with cells purified from peripheral blood, lymph nodes (LNs) are likely a key place where CD56^bright^ NK cells exert their regulatory function (3), since they preferentially home to parafollicular T cell areas (4) where immune responses develop. In addition to CD56^bright^ NK cells, the major NK cell subset in peripheral blood, CD56^dim^ NK cells, which derive from CD56^bright^ NK cells and are more differentiated, also exert regulatory functions as discussed below.

### Interactions between Regulatory NK Cells and Innate Immune Cells

CD56^bright^ NK cells express receptors for cytokines such as interleukin (IL)-12, IL-15, and IL-18 (5–7), which are produced by activated antigen-presenting cells (APCs). These cytokines can trigger proliferation of CD56^bright^ NK cells and their production of molecules such as IFN-γ, IL-10 and IL-13, TNF-β, and GM-CSF (2). In this context, Ferlazzo et al. demonstrated that dendritic cells (DCs) are a key source of IL-12 and IL-15 for activation of CD56^bright^ NK cells (8), and we have shown that DC-derived IL-27 can modulate proliferation and function of these cells (9). Thus, APCs modulate NK cell functions and phenotype (10–13). Infections most likely modulate the function of CD56^bright^ NK cells indirectly through APCs, because co-culturing CD56^bright^ with APCs activated via TLR4 (macrophages, DC) or TLR9 (plasmacytoid DCs) stimulates their proliferation and cytokine production (2, 8, 14, 15). Conversely, activated NK cells modulate the function of APCs: they stimulate monocytes to produce TNF-α (16) and kill immature DCs in a process called DC editing (17, 18).

### Interactions between Regulatory NK Cells and Adaptive Immune Cells

**Figure 1:**
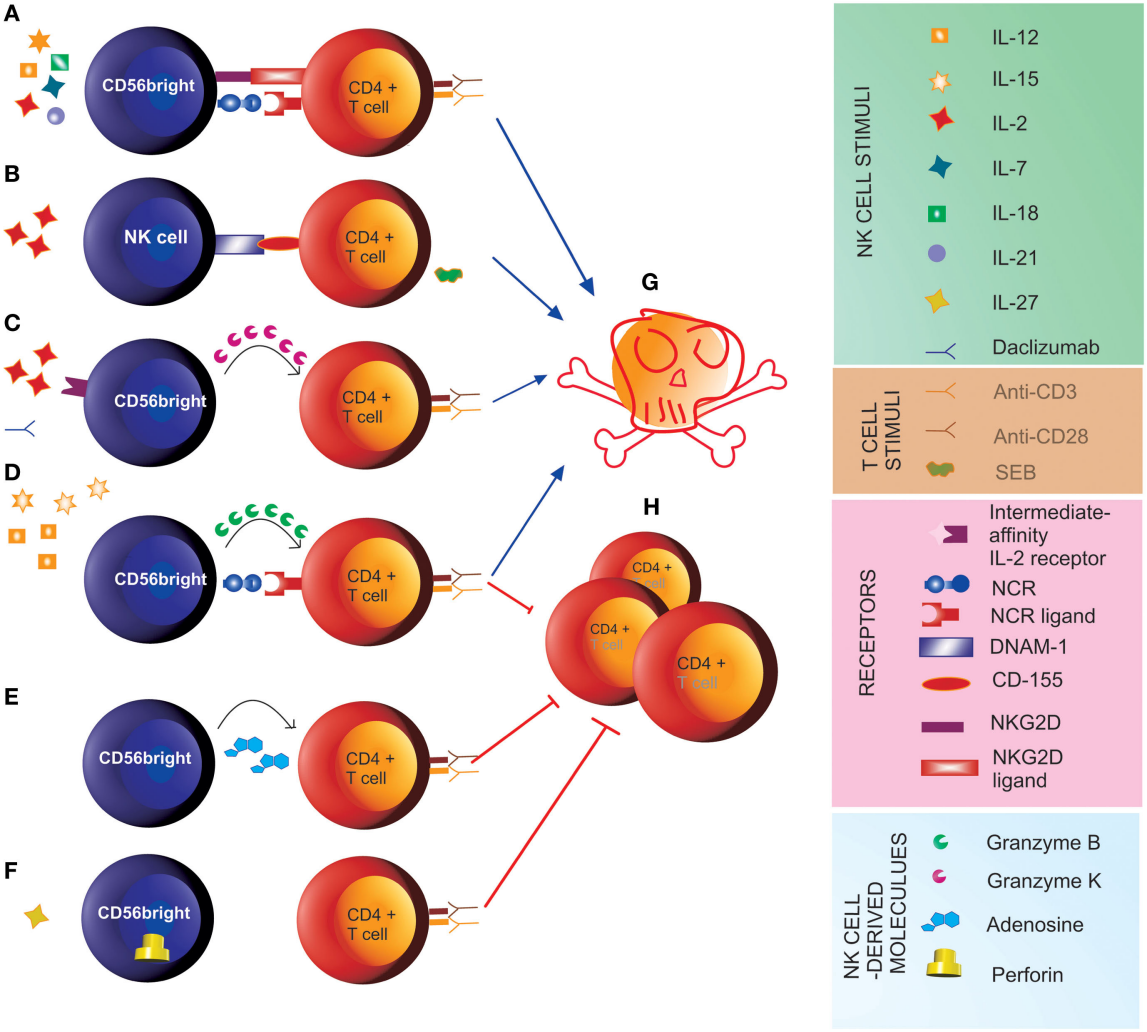
**Natural killer (NK) cell-mediated control of T cell responses**. **(A)** Stimulus of CD56^bright^ with IL-2, IL-7, IL-12, IL-15, IL-18, and IL-21 induces cytotoxicity **(G)** toward previously activated autologous CD4^+^ T cells through the engagement of NKG2D and the natural cytotoxicity receptors (NCRs) (27). **(B)** Pre-activation of NK cells with IL-2 and of autologous CD4^+^ T cells with staphylococcal enterotoxin B (SEB) induces cytotoxicity of NK cells toward autologous T cells **(G)** through engagement of the activating receptor DNAM-1 on NK cells and its ligand CD155 on T cells (21). **(C)** In the presence of the anti-IL-2Rα monoclonal antibody daclizumab, IL-2 signal through the intermediate-affinity receptor induces cytotoxicity of CD56^bright^ NK cells toward autologous activated CD4^+^ T cells **(G)** involving the transfer of granzyme K to target cells (23). **(D)** The pro-inflammatory cytokines IL-12 and IL-15 induce anti-proliferative **(H)** and cytotoxic function of CD56^bright^ NK cells toward CD4^+^ T cells undergoing activation through engagement of the NCRs NKp30 and NKp46 and release of granzyme B (25). **(E)** CD56^bright^ suppress proliferation of autologous CD4^+^ T cells **(H)** by releasing adenosine (26). **(F)** IL-27 induces suppressor function of CD56^bright^ NK cells toward autologous CD4^+^ T cells **(H)**, which is associated with increased perforin content (9).

Natural killer cells also interact with adaptive effector cells. IFN-γ secreted by CD56^bright^ NK cells in response to T cell-derived IL-2 has been demonstrated to stimulate T cells in LNs (4). Along this line, increased local bioavailability of IL-2 by blocking the IL-2Rα chain (CD25) on recently activated T cells upon treatment with daclizumab is associated with expansion and activation of CD56^bright^ NK cells in multiple sclerosis (MS) patients (19–21). Indeed, while T cells express the high-affinity form of the IL-2 receptor, which comprises CD25, CD56^bright^ NK cells express both high-affinity and intermediate-affinity (not comprising CD25) forms of the IL-2 receptor (20, 22). Thus, upon daclizumab treatment, NK cells are stimulated through binding of IL-2 to their intermediate-affinity receptor. This results in control of T cell activation through direct killing (19, 21), which, for the CD56^bright^ subset, involves release of cytotoxic granzyme K (23). Furthermore, IL-27-stimulated CD56^bright^ NK cells have been shown to suppress the proliferation of autologous CD4^+^ T cells in a contact-dependent manner associated with increased perforin content (9). CD56^bright^ NK cells, stimulated with the pro-inflammatory cytokines IL-12 and IL-15, prevent autologous CD4^+^ T cell proliferation through a cytotoxic mechanism involving the engagement of the natural cytotoxicity receptors (NCRs), such as NKp30 and NKp46 (24), on NK cells and the release of granzyme B (25). CD56^bright^ NK cells were also shown to inhibit proliferation of autologous CD4^+^ T cells by secreting the immunosuppressive molecule adenosine. Inhibition of CD38 (“ADP ribosyl-cyclase”), an enzyme involved in the production of adenosine, restored proliferation of T cells in the presence of CD56^bright^ NK cells (26). While these studies described the effects of CD56^bright^ NK cells on T cells undergoing activation, others reported direct cytotoxicity of CD56^bright^ NK cells on previously activated T cells. Nielsen and coauthors found that killing of pre-activated T cells by CD56^bright^ NK cells involves the activating receptors NKG2D, LFA-1, and TRAIL and is enhanced when blocking NKG2A (27). Another study demonstrated that both CD56^bright^ and CD56^dim^ NK cells kill autologous antigen-activated CD4^+^ T cells through engagement of DNAM-1 and 2B4 and their cognate receptors CD155 and CD48, respectively (21). These and other studies reveal that different stimuli activate NK cells toward cytotoxicity and/or suppression of T cell proliferation (Figure 1).

## Possible Role of NK Cells in MS and Its Animal Model

Multiple sclerosis is an autoimmune disease of the central nervous system (CNS) characterized by an attack of the myelin sheath that surrounds and protects CNS axons by autoreactive T cells. Its murine model, experimental autoimmune encephalomyelitis (EAE), is triggered by active immunization with myelin antigens or transfer of activated autoreactive myelin-specific T cells to naïve recipients. Until recently, defects in regulatory mechanisms had only been described in MS in cells of the adaptive compartment (28).

Conflicting data on a beneficial vs. detrimental role of NK cells in EAE have been published (29–31), but studies on regulatory NK cells in mice are difficult to translate into humans, because murine NK cells do not express CD56 and the murine counterparts of CD56^bright^ and CD56^dim^ subsets have not been identified with certainty.

Enhancing regulatory features of NK cells ameliorates the disease course of EAE. In particular, NK cells expressing NKG2A (which is expressed by all CD56^bright^ NK cells in humans) were shown to decrease CNS inflammation by killing T cells and microglial cells, when the interaction between NKG2A and its ligand Qa-1 (the murine equivalent to the human HLA-E) expressed on the target cells was blocked by antibodies specific for either antigen (32, 33). Decreased expression of Qa-1 on microglial cells upon CNS inflammation rendered them more sensitive to NKG2A^+^ NK cell-mediated lysis. Importantly, enrichment of NK cells through treatment with IL-2 coupled with IL-2 mAb (“IL-2 complexes”) (34) ameliorated EAE (35). A recent work from the same group has shed light on the differential effects of NK cells in early vs. late stages of EAE and possibly MS (36), which may depend on their interactions with neural stem cells (NSCs). Indeed, Liu et al. found that, in MS and EAE brains, NK cells are in contact with NSCs and that, in EAE NSCs released IL-15 upon contact with NK cells, thereby supporting NK cell proliferation and survival; in turn, NK cells killed NSCs, particularly during the late stages of EAE, as a result of reduced expression of Qa-1 on NSCs. Accordingly, removal of NK cells during the late phase of EAE reduced disease severity (36). These observations suggest that cytotoxicity of NK cells may be a double-edged sword in EAE, with NK cells attenuating inflammation in the acute phase of disease by killing immune cells (T cells, microglial cells), but impairing potential repair during the late stage by killing NSCs.

While Liu et al. did not ascertain whether NK cells in contact with NSCs belonged to the CD56^bright^ or CD56^dim^ NK subset (36), others analyzed the phenotype of NK cells in the cerebrospinal fluid (CSF). The majority of intrathecal NK cells in healthy individuals, MS patients and patients with other neurological diseases are CD56^bright^ NK cells (21, 37, 38), suggesting that CSF enrichment in CD56^bright^ NK cells is not MS specific, but rather CNS specific (37). This may reflect organ-specific, rather than blood-specific, tropism of CD56^bright^ NK cells (39). The recently discovered lymphatic vessels in the brain (40, 41) may be the route of entry for CD56^bright^ NK cells, which were shown to circulate in the lymph (42). Of note, a higher migratory capacity of CD56^bright^ compared to CD56^dim^ NK cells was observed in a model of the human blood–brain barrier (BBB) (21).

Given the evidence that CD56^bright^ NK cells are a regulatory population of the IS in healthy individuals, we have explored their function in untreated MS patients or patients with clinically isolated syndrome suggestive of MS (25). The number of CD56^bright^ NK cells was similar in MS patients and healthy subjects (HS). However, upon stimulus with pro-inflammatory cytokines, CD56^bright^ NK cells from MS patients suppressed much less efficiently the proliferation of autologous CD4^+^ T cells compared to those from HS. This was associated with an increased expression of HLA-E on CD4^+^ T cells in MS and was reverted by blocking HLA class I on T cells, suggesting that the cytotoxic function of CD56^bright^ NK cells on their targets is inhibited through binding of HLA-E on T cells to the NK cell inhibitory ligand NKG2A. HLA-E is a non-classic major HLA class I molecule expressed by immune cells and, outside the immune compartment, by endothelial cells, which release its soluble form upon inflammation (43). HLA-E upregulation was found in MS CNS within white matter lesions, in endothelial cells and astrocytes (44, 45). Similarly, Morandi et al. detected increased levels of soluble HLA-E in the CSF and expression of HLA-E within immune cells and neural cells in MS plaques, which correlated with decreased NK cell cytotoxicity (46). The causes of such upregulation are, as yet, unknown. Thus, an impairment of CD56^bright^ NK cell immunoregulatory function in MS may occur not only in the periphery but also within the CNS. Recently, we also described decreased cytolytic activity of NK cells in MS as a consequence of reduced upregulation of CD155 on T cells after activation, concomitantly with a reduced NK cell surface expression of DNAM-1 (21). These studies point toward a resistance of T cells to NK cell suppressive functions rather than an intrinsic defect in the NK cells in MS.

The relevance of the immunoregulatory function of NK cells in MS is emphasized by studies from the group of Takashi Yamamura, who described a particular peripheral NK phenotype to be characteristic of MS patients in remission, with increased production of the anti-inflammatory cytokine, IL-5 (“NK2” cells), and high expression of CD95 (47), which inhibited the production of IFN-γ by Th1 clones (48). Among these patients, a further division of NK2 cells as CD11c-high (not producing IL-5) and CD11c-low (producing IL-5) subsets identified CD11c-high patients at risk of relapse (49), suggesting that CD11c + NK cells are pro-inflammatory. In another study, a subpopulation of NK cells characterized by low expression of CD8, a phenotype associated with CD56^bright^ NK cells, was observed to be reduced in untreated patients with relapsing-remitting MS (50).

Previous infections may influence the development of MS (51). Interestingly, infections not only activate NK cells but also shape NK cell functions (52–54). Thus, cytomegalovirus (CMV) induces expansion of NK cells that produce IL-10 in mice, to prevent excess of activation of CD8 T cells (55, 56). In humans, CMV infection is associated with expansion of terminally differentiated NK cells bearing the NKG2C receptor and has been implicated both in MS etiology and/or “protection” (57). In this context, Martinez-Rodriguez et al. explored the expression of NKG2C on NK cells from MS patients and controls, in relation to their CMV^+^ serostatus and to the NKG2C genotype, finding that the expansion of NKG2C^+^ NK cells in CMV^+^ patients was associated to lower risk of disease progression, suggesting that CMV may exert a beneficial influence on MS, either through expansion of NKG2C^+^ NK cells or through other mechanisms (58). Differently to CMV, infection with Epstein–Barr virus, which has also been associated with an increased risk of MS, expands early differentiated NKG2A + CD56^dim^NK cells (59, 60), but whether such cells have any role in the pathogenesis of MS is unknown.

## The Impact of MS Therapies on NK Cells

**Figure 2:**
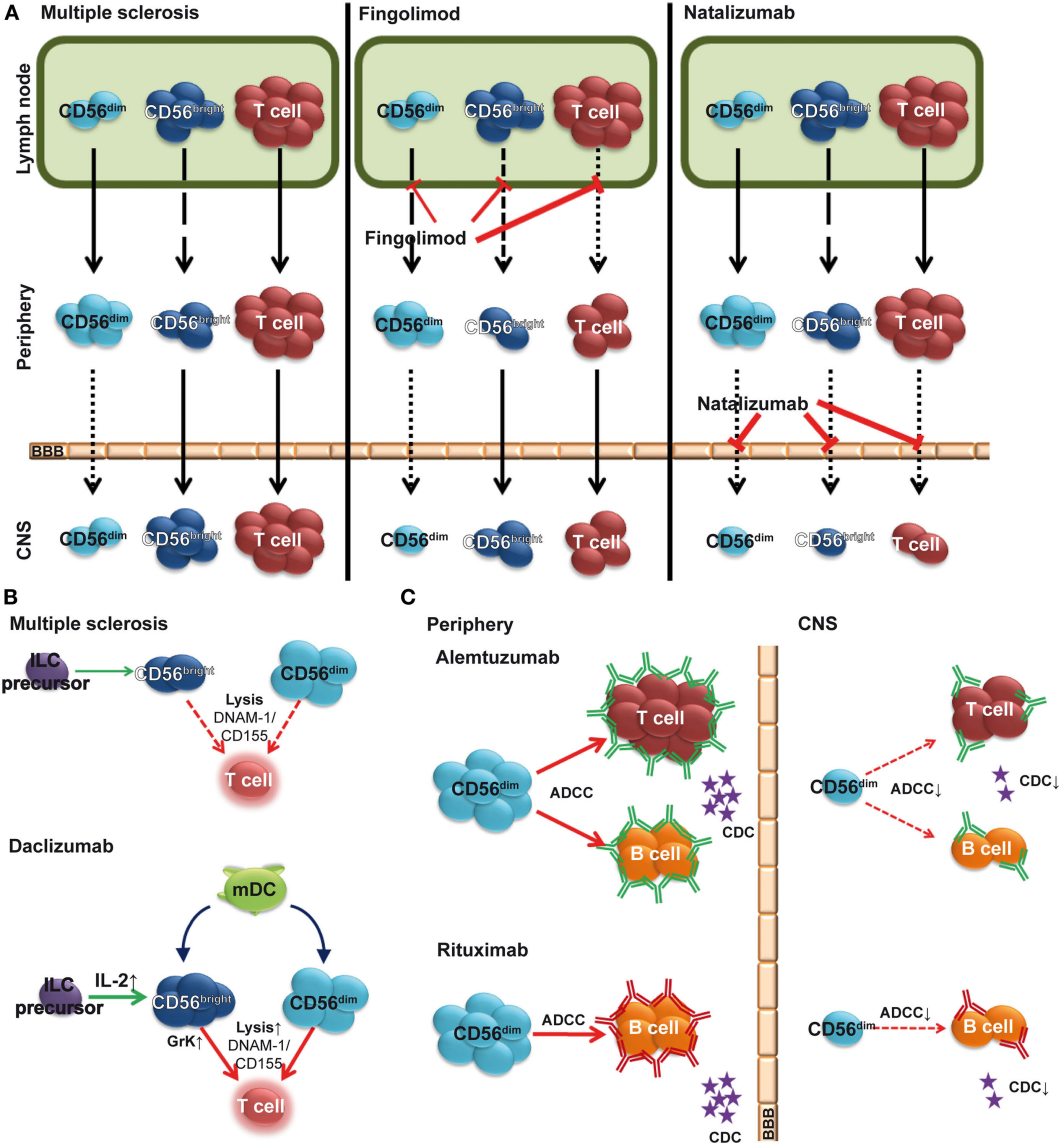
**Impact of multiple sclerosis (MS) therapies on the natural killer (NK) cell compartment**. **(A)** Fingolimod (FTY720) inhibits egress of CD56^bright^, and to a lower degree, CD56^dim^ NK cells from the lymph node (LN), resulting in a relative increase of the latter subset in the periphery (middle). Natalizumab inhibits transmigration of lymphocytes including NK cells across the blood–brain barrier (BBB). **(B)** Elevated levels of IL-2 in daclizumab-treated patients promote differentiation and expansion of CD56^bright^ NK cells. Daclizumab both boosts NK cell cytolytic function in a DC-dependent manner and renders antigen-activated T cells more sensitive toward NK-mediated lysis, thus restoring defective NK-mediated control of T cell activity in MS. **(C)** In addition to complement-dependent cytotoxicity (CDC), alemtuzumab (top) and rituximab (bottom) use CD56^dim^ NK cell-mediated antibody-dependent cellular cytotoxicity (ADCC) to deplete T and/or B cells in peripheral blood. However, in the CSF, sparseness of CD56^dim^ NK cells, reduced levels of complement proteins, and lack of antibodies crossing the BBB limit local immune-modulating efficacy [**(C)** adapted from Ref. (66)].

In addition to first-line MS therapies, interferon beta (IFN-β) and glatiramer acetate, novel immune-modulating therapies such as the anti-inflammatory dimethyl fumarate, the T cell proliferation inhibitor teriflunomide, the migration inhibitors natalizumab and fingolimod (FTY720), the IL-2 receptor-modulating daclizumab, and the immune cell-depleting alemtuzumab are now available for treatment of MS (61, 62). Many of these immune-modulating biologicals alter the NK cell compartment (Figures 2A,B) by increasing NK cell frequencies (Figure 2A) and/or NK-mediated immune regulatory functions (Figure 2B) (61, 63), which points to an immune-protective role of NK cells in MS. Furthermore, antibody-dependent cell-mediated cytotoxicity (ADCC) by CD56^dim^ NK cells has been proposed as an essential therapeutic mechanism in alemtuzumab-mediated T and B cell depletion as well as rituximab-mediated B cell depletion (Figure 2C) (64, 65).

One therapeutic approach in MS is the reduction of inflammatory lesions by inhibiting infiltration of autoreactive lymphocytes into the CNS. While the humanized monoclonal antibody (hMA) anti-CD49d (alpha 4 integrin) natalizumab prevents transmigration of circulating lymphocytes across the BBB (67), the sphingosine 1-phosphate receptor (S1P) modulator FTY720 reduces CNS inflammation in MS (68) indirectly by preventing lymphocyte egress from LNs (69). A natalizumab-induced increase of total NK cells and CD56^bright^ NK cells in blood concomitantly with reduced NK cell numbers in CSF (Figure 2A) (21, 70, 71) suggests CD49d-dependent transmigration of NK cells into the CNS. Trafficking of NK cells in steady state and inflammatory conditions requires S1P (72) and decreased numbers of circulating CD56^bright^ NK cells have been observed 6 h after FTY720 treatment (73) (Figure 2A). Long-term treatment also resulted in reduced numbers of both NK cell subsets in the CSF (own observations). Despite reduced NK cell numbers, a relative increase in peripheral and intrathecal NK cell subsets within the lymphocyte compartment (74) indicates that FTY720 inhibits NK cell emigration less than that of other lymphocytes (Figure 2A). This might be due to the fact that egress of NK cells from LNs is regulated through both S1P_1_ and S1P_5_ (72, 75), whereas other lymphocytes use only S1P_1_ (76). Since S1P_1_ and S1P_5_ seem to trigger the activation of distinct intracellular signal transduction pathways, it has been suggested that S1P_5_ might be less susceptible to FTY720 than S1P_1_ (72). Along this line, a higher expression of S1P_5_ on CD56^dim^ NK cells than on CD56^bright^ ones (72) might explain the relative increase of the CD56^dim^ NK cell subset (77). While treatment of MS patients with FTY720 alters NK cell trafficking, it has no impact on cytokine secretion or cytolytic function of NK cells (77).

In contrast, treatment with daclizumab affects both peripheral (20, 78) and intrathecal CD56^bright^ NK cell numbers (79), as well as their immunoregulatory function (19, 21) (Figures 1 and 2B). Daclizumab is a recently approved hMA directed against IL-2Rα, which showed enhanced efficacy in MS compared to IFN-β [DECIDE trial (80)]. Daclizumab enhances endogenous mechanisms of immune tolerance by reducing early T cell activation (81), expanding CD56^bright^ NK cells (20, 78), while reducing lymphoid tissue-inducer cells (82), and restoring defective NK cell-mediated control of T cell activity in MS (19, 21). The mechanism of the effect of daclizumab on CD56^bright^ NK cells has been discussed in chapter 1.3 (Figure 2C). Daclizumab both boosts NK cell cytolytic function in a DC-dependent manner and renders antigen-activated T cells more sensitive toward NK-mediated lysis, thus restoring defective NK cell-mediated control of T cell activity in MS (19, 21) (Figure 2B).

Alemtuzumab is a hMA directed against the cell surface molecule CD52, which demonstrated a high clinical efficacy in MS (83, 84). CD52 is highly expressed on T and B cells and to a lower degree on NK cell subsets (85, 86). Accordingly, a relative increase of circulating NK cells with increased numbers of CD56^bright^ NK cells was observed 6 months after alemtuzumab therapy, whereas the CD56^dim^ subset remained unaltered. However, there was no change in NK cell cytolytic function (87). ADCC is mediated via the FcγRIII (CD16)-expressing CD56^dim^ NK cell subset (88). Since intrathecal CD56^dim^ NK cells are sparse (21, 89), the therapeutic efficacy of alemtuzumab within the CNS might be limited (Figure 2C). Along this line, insufficient disease inhibition in progressive MS by intrathecal application of rituximab was proposed to be due to low numbers of CD56^dim^ NK cells and reduced levels of complement proteins within the CNS (66). Further studies are required to shed more light on NK cell-mediated ADCC as a mechanism of action of human monoclonal antibody-mediated depleting therapies in MS.

## Summary/Outlook

NK cells are important players in controlling T cell activation in CNS autoimmunity, and impaired immune regulatory function of NK cells might be one of the driving forces in the pathogenesis of MS. Thus, a better understanding of the underlying mechanisms of NK cell-mediated regulation of T cell activation might help to improve treatment strategies in MS.

## Author Contributions

CG wrote chapter 3, conceptualized Figure 2, revised the manuscript, and approved the final version. AS-M drew Figure 2, revised the manuscript, and approved the final version. HW wrote chapter 3, revised the manuscript, and approved the final version. EM critically reviewed the manuscript for important intellectual content and approved the final version. NKdeR conceptualized Figure 1, critically reviewed the manuscript for important intellectual content, and approved the final version. AU critically reviewed the manuscript for important intellectual content and approved the final version. AL organized the sections of the manuscript, wrote chapters 1 and 2, conceptualized and drew Figure 1, revised the manuscript, and approved the final version.

## Conflict of Interest Statement

CG received speaker honoraria and travel expenses for attending meetings from Genzyme, Novartis Pharma GmbH, and Bayer Health Care. AS-M has no financial disclosures. HW received compensation for serving on Scientific Advisory Boards/Steering Committees for Bayer Healthcare, Biogen, Merck Serono, Novartis, and Sanofi-Genzyme. He also received speaker honoraria and travel support from Bayer vital GmbH, Bayer Schering AG, Biogen, CSL Behring, Fresenius Medical Care, Glaxo Smith Kline, GW Pharmaceuticals, Lundbeck, Merck Serono, Omniamed, Novartis, and Sanofi-Genzyme. He received compensation as a consultant from Biogen, Merck Serono, Novartis, and Sanofi-Aventis. He received research support from Bayer Vital, Biogen, Genzyme, Merck Serono, Novartis, Sanofi-Genzyme Germany, and Sanofi US. EM has no financial disclosures. NKdeR has no financial disclosures. AU received grants and contracts from Fondazione Italiana Sclerosi Multipla (FISM), Novartis, Fondazione Cariplo, and Italian Ministry of Health; received honoraria or consultation fees from Biogen, Roche, Teva, Merck Serono, Genzyme, and Novartis. He is a member of a company advisory board, board of directors, or other similar groups for Roche and Genzyme. AL received honoraria for speaking by Biogen, Novartis, and Teva, consulting fees by Merck Serono, Sanofi-Genzyme, and Novartis, and funding for travel from Teva, Merck Serono, Biogen, and Novartis.
